# Initiation of antihypertensive monotherapy and incident fractures among Medicare beneficiaries

**DOI:** 10.1186/s40621-017-0125-8

**Published:** 2017-10-18

**Authors:** Jennifer L. Hargrove, Yvonne M. Golightly, Virginia Pate, Carri H. Casteel, Laura R. Loehr, Stephen W. Marshall, Til Stürmer

**Affiliations:** 1 Gillings School of Global Public Health, The University of North Carolina at Chapel Hill, Chapel Hill, NC USA; 2 College of Public Health, The University of Iowa, Iowa City, IA USA

**Keywords:** Fractures, Older adults, Epidemiology, Antihypertensive initiation

## Abstract

**Background:**

Research suggests antihypertensive medications are associated with fractures in older adults, however results are inconsistent and few have examined how the association varies over time. We sought to examine the association between antihypertensive class and incident non-vertebral fractures among older adults initiating monotherapy according to time since initiation.

**Methods:**

We used a new-user cohort design to identify Medicare beneficiaries (≥ 65 years of age) initiating antihypertensive monotherapy during 2008–2011 using a 20% random sample of Fee-For-Service Medicare beneficiaries enrolled in parts A (inpatient services), B (outpatient services), and D (prescription medication) coverage. Starting the day after the initial antihypertensive prescription, we followed beneficiaries for incident non-vertebral fractures. We used multinomial logistic regression models to estimate propensity scores for initiating each antihypertensive drug class. Using these propensity scores, we weighted beneficiaries to achieve the same baseline covariate distribution as beneficiaries initiating with angiotensin-converting enzyme inhibitors. Lastly, we used weighted Cox proportional hazard models to estimate hazard ratios (HRs) of having an incident fractures according to antihypertensive class and time since initiation.

**Results:**

During 2008–2011, 122,629 Medicare beneficiaries initiated antihypertensive monotherapy (mean age 75, 61% women, 86% White). Fracture rates varied according to days since initiation and antihypertensive class. Beneficiaries initiating with thiazides had the highest fracture rate in the first 14 days following initiation (438 per 10,000 person-years, 95% confidence interval (CI): 294–628; HR: 1.40, 0.78–2.52). However, beneficiaries initiating with calcium channel blockers had the highest fracture rate during the 15–365 days after initiation (435 per 10,000 person-years, 95% CI: 404–468; HR: 1.11, 1.00–1.24). Beneficiaries initiating with angiotensin-receptor blockers had the lowest fracture rates during the initial 14 days (333 per 10,000 person-years, 190–546, HR: 0.92, 0.49–1.75) and during 15–365 days after initiation (321 per 10,000 person-years, 287–358, HR: 0.96, 0.84–1.09).

**Conclusion:**

The association between antihypertensives and fractures varied according to class and time since initiation*.* Results suggest that when deciding upon antihypertensive therapy, clinicians may want to consider possible fracture risks when choosing between antihypertensive drug classes.

**Electronic supplementary material:**

The online version of this article (10.1186/s40621-017-0125-8) contains supplementary material, which is available to authorized users.

## Background

Fractures are one of the most common fall-related injuries for adults over the age of 65 (DeGrauw et al. 2016). In older adults, fractures are associated with high medical costs, loss of independence, and an increased risk of mortality (Stevens et al. 2006; Leibson et al. 2002; Tinetti and Williams 1997; Haentjens et al. 2010). Older adults are at greater risk of fractures due to decreased bone mineral density (BMD), decreased physical activity, and increased comorbidities (Marks et al. 2003). Comorbidities such as cardiovascular disease, osteoporosis, Parkinson’s disease, and diabetes can increase fracture risk (Woolf and Åkesson 2003; Lee et al. 2015). Medications increasing the risk of fractures include opioids, benzodiazepines, antidepressants, antiepileptics, and skeletal muscle relaxants (Fraser et al. 2014; By the American Geriatrics Society 2015 Beers Criteria Update Expert Panel 2015; Takkouche et al. 2007). Recently, research suggests antihypertensives may increase the risk of fall-related injury by as much as 11% among older adults (Butt and Harvey 2015; Tinetti et al. 2014).

Antihypertensives are associated with fractures through medication-related adverse events and by interactions with BMD (Berry and Kiel 2014). In the first few weeks after initiation, antihypertensives can increase the risk of falls and subsequent fractures due to medication-related adverse events, such as orthostatic hypotension (Butt and Harvey 2015; Zia et al. 2015). Orthostatic hypotension is defined as a decrease in blood pressure upon standing (Gupta and Lipsitz 2007), and is associated with an elevated risk of falls and fractures among hypertensive adults (Gangavati et al. 2011). Additionally, antihypertensives have been found to impact BMD in observational and clinical studies. For instance, thiazide diuretics reduce urinary calcium secretion and can stimulate osteoblasts potentially providing a protective effect for fractures (Ghosh and Majumdar 2014; Ruths et al. 2015; Solomon et al. 2011). Angiotensin-receptor blockers and angiotensin-converting enzyme inhibitors are believed to impact BMD by inhibiting bone turnover caused by the renin-angiotensin-aldosterone system (Ghosh and Majumdar 2014).

Prior research has found inconsistent results regarding the strength and direction of the association between antihypertensives and fractures (Butt and Harvey 2015; Zang 2013). Some studies have found antihypertensives increase the risk of fractures (Tinetti et al. 2014; Choi et al. 2015; Butt et al. 2012), while others have found no association (Fraser et al. 2014), or a protective association with fractures (Ruths et al. 2015; Solomon et al. 2011; Rejnmark et al. 2006). Few studies have examined the initial increased risk of fractures associated with starting antihypertensive therapy, and how the association between antihypertensives and fractures varies time. Therefore, we sought to examine the association between antihypertensives and incident non-vertebral fractures within the first year of initiation among Medicare beneficiaries. Specifically, we hypothesized that fractures in the first 2 weeks would be due to medication-related adverse events and fractures that occurred between 2 weeks and 1 year of use would be due to possible BMD effects.

## Methods

### Data source

We used a 20% nationwide, random sample of fee-for-service Medicare beneficiaries who were enrolled at least 1 month in Medicare Parts A (inpatient care), B (outpatient care), and D (prescription drugs) coverage between 2007 and 2011. Data were obtained under a data use agreement established with the Centers for Medicaid and Medicare Services (CMS) and the University of North Carolina at Chapel Hill (UNC-CH). The study protocol was approved by UNC’s Non-Biomedical Institutional Review Board.

### New users of antihypertensive monotherapy

The study cohort consisted of all new users of antihypertensive medication initiating during 2008–2011 who were continuously enrolled in Medicare Parts A, B, and D for at least 12 months prior to initiation. New use was defined as not having a prior prescription of the following antihypertensive medications in the last 12 months: angiotensin-converting enzyme inhibitors (ACE), angiotensin-receptor blockers (ARB), beta blockers (BB), calcium channel blockers (CCB), or thiazide diuretics (THZ). We limited the cohort to beneficiaries initiating with monotherapy (e.g., one class of antihypertensive drug) since we were interested in examining differences in effect by drug class. Loop diuretics were excluded from the exposure definition since these drugs are typically prescribed for different indications and are a marker of increased short-term mortality in to older adults (Glynn et al. 2001).

We excluded beneficiaries who were originally eligible for Medicare due to end stage renal disease or disability and those beneficiaries with prior nursing home stays since not all medications dispensed in the nursing home are captured in Part D data. Additionally, we excluded beneficiaries who had a previous diagnoses for tremors or congestive heart failure since these health conditions could result in being prescribed antihypertensives. Since we were interested in capturing incident fractures and since prior falls can increase future fall and fracture risk (Pohl et al. 2014), we excluded beneficiaries who had prior falls or fractures. Finally, we required a second antihypertensive fill within 30 days of the end of the index drug’s days supply to exclude those beneficiaries who filled the first prescription and never returned (Additional file 1: Figure S1). We did not exclude beneficiaries with chronic kidney disease or diabetes, despite these being indications for certain antihypertensive drug classes, however we did conduct sub-analyses excluding these beneficiaries (see Statistical Analysis).

### Initiation of antihypertensive therapy

Prescription medication data were identified using Medicare Part D. Using fill dates, we identified the date of the initial prescription for antihypertensive therapy (index date). Antihypertensive medications were identified using National Drug Codes and generic drug names (Additional file 1: Table S1). These specific drug classes were chosen based on the current recommendations for hypertension treatment in older adults (James et al. 2014).

### Incident non-vertebral fractures

Incident non-vertebral fractures within 12 months of initiating antihypertensive monotherapy were our primary outcome. Starting the day after the index date, we followed beneficiaries until the first fracture event. Fractures were identified using validated diagnostic and procedure codes in Medicare Parts A and B (Additional file 1: Table S2) (Ray et al. 1992). We chose to examine fractures instead of falls since the accuracy of fall reporting varies by state and has low specificity in claims data (Annest et al. 2008). We excluded fractures that had a corresponding external cause-of-injury relating to motor vehicle crashes (E810-E825) and those that occurred on the index date.

In sub-analyses, we grouped fractures according to the anatomical location to distinguish between fractures that were likely related to low BMD. Low BMD fractures, or osteoporotic fractures, are typically defined as fractures occurring at the hip, radius, or vertebrae (Johnell and Kanis 2005). Since incident vertebral fractures are not well captured in claims data (Curtis et al. 2009), we defined probable low BMD fractures as any fracture event involving the hip or radius. All other non-vertebral fractures were classified as probable normal BMD fractures.

### Risk factors for fractures

Covariates were selected based on the previous literature (Woolf and Åkesson 2003; Lee et al. 2015; By the American Geriatrics Society 2015 Beers Criteria Update Expert Panel 2015; Zia et al. 2015; Nurminen et al. 2013) and were defined based on claims during the 12 months prior to initiation (Additional file 1: Table S3). Covariates included: demographics (age, gender, and race), concurrent medication use and prior use of medications associated with fractures (loop diuretics, antiarrhythmics, antidepressants, antiepileptics, anxiolytics, benzodiazepines, bisphosphonates, antipsychotics, skeletal muscle relaxants, opioids, and hypnotics), codes for chronic comorbidities associated with fracture risk (diabetes, chronic kidney disease, Parkinson’s disease, Alzheimer’s disease, osteoporosis, arrhythmia, osteoarthritis, rheumatoid arthritis, stroke, myocardial infarction, hypertension, orthostatic hypotension, syncope, dementia, urinary incontinence, dyslipidemia, and obesity), frailty predictors, and prior hospital admissions. Concurrent medication use was defined as the number of distinct drug prescriptions filled in the 14 days prior to antihypertensive initiation. As a proxy for sociodemographic status, we identified whether beneficiaries were eligible for the Medicare low-income subsidy (LIS) program. LIS offers medication at a reduced cost for beneficiaries that are eligible due to income, family size, and household resources. We included the frailty index score as a proxy measure of frailty (Faurot et al. 2015). Additionally, we examined the prevalence of factors positively (ambulance transfer, wheelchair/walker use, home oxygen use, hospital bed, difficulty walking, and vertigo) and inversely (cancer screenings) associated with limitations in activities of daily living (Faurot et al. 2015).

### Statistical analysis

Descriptive statistics were used to compare baseline covariates according to antihypertensive class initiated on the index date. We estimated propensity scores (PS) using multinomial logistic regression models. PSs estimated the probability of receiving an ACE vs. other classes of antihypertensive drugs adjusting for all baseline covariates. Standardized mortality ratio (SMR) weighting was used to control for confounding due to differences in propensity scores. We weighed beneficiaries of each drug class to achieve the same baseline covariate distribution as beneficiaries receiving an ACE. Therefore, beneficiaries initiating with ACEs were assigned a weight of one and all others were assigned a weight that was the ratio of the PS to one minus the PS (Stürmer et al. 2006). ACEs were used as the referent since they were the most commonly prescribed drug class (Li et al. 2014). To examine the effectiveness of PSs and SMR weights to correctly balance baseline covariates between beneficiaries, we examined the means and proportions of baseline covariates before vs. after SMR weighting. If the PSs are correctly specified, there should be little difference between the mean and proportion of baseline covariates across antihypertenisves classes.

Incident fracture rates and corresponding 95% confidence intervals (CIs) were defined as the total number of incident fractures by the total person-years at risk. Person-years at risk was defined as the total number of days at risk for fractures divided by 365.25. To account for censoring, we used SMR-weighted Cox proportional hazard models to estimate hazard ratios (HRs) and 95% CIs of incident fractures for each drug class initiated on the index date versus receiving an ACE according to days since initiation of therapy, 1–14 days and 15–365 days. We separated time since initiation to distinguish between fractures likely related orthostatis and fractures that may be the result of possible BMD effects. CIs were calculated using robust standard errors to account for SMR weights. We used a ‘first-treatment-carried-forward’ analysis to avoid introducing confounding by indication since antihypertensive adherence varies, and beneficiaries who remain adherent may differ from the majority of hypertensive patients (Hargrove et al. 2017). Using this analysis, beneficiaries contributed person-time at risk until they had an incident fracture or until the end of the follow-up (death, disenrollment from Medicare, or December 31, 2012), whichever came first. SMR weighted Kaplan-Meier curves were used to graph the proportion of beneficiaries without fracture events according to time since initiation. For our secondary analysis when we classified fractures according to probable low vs. normal BMD fractures, if a beneficiary had a fracture event before the event of interest (e.g., normal BMD fracture before a low BMD fracture), beneficiaries were censored at the date of the first fracture event.

### Sensitivity analyses

To assess the robustness of our analysis decisions, we performed sensitivity analyses. First, we repeated the analysis using an ‘As-treated’ analysis design. In the ‘As-treated’ analysis, follow-up additionally ended when beneficiaries switched antihypertensive therapy (e.g., switched to another antihypertensive class or started combination therapy), or discontinued use (e.g., failed to fill another prescription 30 days after the end of the last drugs’ days supply). Second, we repeated the analysis extending the first follow-up period to 30 days since the time it takes for blood pressure to stabilize after antihypertensive initiation is not known. Third, we repeated the primary analysis excluding beneficiaries who initiated therapy using a brand antihypertensive medication versus a generic antihypertensive medication. Generic medications are less prone to sample use and are thus less prone to have started antihypertensive therapy before the first dispensed prescription (Hampp et al. 2016). Fourth, since chronic kidney disease and diabetes can impact physicians’ choice of antihypertensive class prescribed, we repeated the analysis removing any beneficiaries with these chronic conditions. Lastly, we repeated the analysis removing beneficiaries prescribed loop diuretics during the baseline period since these medications may be a marker for overall worse health and greater risk of morality (Glynn et al. 2001).

All statistical analyses were conducted using SAS Version 9.4 (Cary, NC).

## Results

Between 2008 and 2011, 122,629 Medicare beneficiaries initiated antihypertensive monotherapy. On average beneficiaries were 75 years old, 61% were women, and the majority were White (86%). The most common classes of antihypertensives prescribed were ACEs (33%), BBs (30%), and CCBs (15%). Before SMR weighting, demographics, diabetes, chronic kidney disease, cardiovascular disease (e.g., arrhythmia, stroke, hypertension, and dyslipidemia), ambulance transfers, cancer screenings, and prior hospitalizations differed across beneficiaries according to antihypertensive class. After SMR weighting, there was little difference between baseline characteristics according to antihypertensive class (Table 1).

**Table 1:** Characteristics of Medicare beneficiaries initiating antihypertensive monotherapy between 2008 and 2011 (*n* = 122,629)

	ACE *n* = 40,186	ARB *n* = 10,954		BB *n* = 36,972		CCB *n* = 18,411		THZ *n* = 16,106	
	Cohort	Cohort	SMRW	Cohort	SMRW	Cohort	SMRW	Cohort	SMRW
Mean Age, std. (years)	74, 6.7	75, 6.7	74, 12.9	75, 7.0	74, 7.1	76, 7.4	75, 10.0	75, 7.0	75, 10.8
Mean Frailty Index, std	0.1, 0.1	0.1, 0.1	0.1, 0.2	0.1, 0.1	0.1, 0.1	0.1, 0.2	0.1, 0.2	0.1, 0.1	0.1, 0.2
Male	42.0	36.6	41.5	41.7	42.2	37.8	42.1	29.3	42.4
White Race	87.1	79.0	87.3	88.8	86.9	81.8	86.9	86.5	87.2
Low-Income Subsidy	5.3	5.5	5.5	4.6	5.4	5.8	5.4	5.0	5.2
1–2 Meds Filled^a^	61.3	62.4	61.8	58.6	61.4	59.8	62.5	66.9	62.5
3–4 Meds Filled^a^	27.7	26.5	27.4	28.7	27.2	27.1	26.3	24.7	26.5
5 + Meds Filled^a^	11.1	11.2	10.8	12.7	11.4	13.0	11.3	8.5	11.1
Loop Diuretic	5.8	5.9	5.9	7.0	6.1	6.4	6.0	3.7	6.4
Antiarrhythmic	2.6	4.2	2.5	5.4	2.8	4.5	2.7	2.7	3.0
Antidepressant^b^	15.2	14.9	15.2	16.0	15.6	15.9	15.6	15.4	15.5
Antipileptic^b^	7.4	7.3	7.3	8.0	7.5	8.2	7.6	7.7	7.4
Anxiolytic	3.4	3.4	3.5	4.0	3.6	4.1	3.6	3.7	3.5
Benzodiazpene^b^	1.1	1.0	1.1	1.4	1.2	1.3	1.2	1.3	1.1
Bisphosphonate	10.6	12.4	10.6	11.2	10.4	11.6	10.7	13.0	10.8
Antipsychotic^b^	3.5	2.8	3.5	4.2	3.7	4.9	3.7	3.8	3.7
Skeletal Muscle Relaxant^b^	6.2	6.2	6.2	6.5	6.5	6.3	6.4	6.5	6.4
Opioid	27.0	26.2	27.4	30.2	27.2	29.5	27.5	28.0	28.2
Hypnotic^b^	6.1	7.9	6.0	7.5	6.1	7.1	6.2	6.7	6.2
Diabetes	31.6	30.6	31.0	19.0	32.8	18.3	31.4	13.5	32.9
Chronic Kidney Disease	8.7	10.5	8.8	10.6	9.4	13.1	9.3	6.3	9.8
Parkinson’s Disease	1.2	1.1	1.2	1.5	1.2	1.5	1.2	1.3	1.2
Alzheimer’s Disease	3.2	2.4	3.1	3.0	3.4	4.4	3.3	3.1	3.6
Osteoporosis	14.1	17.3	14.2	16.0	14.1	17.4	14.4	16.9	14.6
Arrhythmia	10.5	10.9	10.4	32.9	10.6	25.4	10.6	8.5	11.6
Osteoarthritis	14.4	17.8	14.3	16.1	14.7	15.5	14.9	15.7	15.1
Rheumatoid Arthritis	2.5	3.4	2.5	3.2	2.6	3.0	2.7	2.5	2.6
Stroke	13.7	13.5	13.5	16.6	15.0	16.4	15.1	10.3	14.9
Myocardial Infarction	0.6	0.2	0.6	4.0	0.6	0.6	0.7	0.1	0.7
Hypertension	83.3	88.2	83.5	63.3	84.3	78.6	85.3	76.8	85.0
Orthostatic Hypotension	0.6	0.6	0.7	1.1	0.7	0.9	0.8	0.5	0.6
Syncope	3.8	4.2	3.7	7.4	4.0	6.1	4.2	3.0	4.3
Dementia	5.8	4.2	5.6	5.9	6.1	8.2	6.1	5.6	6.1
Urinary Incontinence	4.7	4.8	4.7	4.9	4.8	5.5	4.9	4.5	4.9
Dyslipidemia	64.8	70.2	64.2	64.0	65.7	58.2	65.3	57.2	65.3
Obesity	4.4	4.2	4.5	3.6	4.6	3.3	4.4	4.1	4.4
Home Oxygen Use	2.4	2.5	2.4	2.5	2.6	5.3	2.3	2.7	2.6
Walker/Wheelchair Use	2.2	2.4	2.2	2.8	2.3	3.3	2.3	2.4	2.5
Hospital Bed Use	0.5	0.5	0.5	0.5	0.5	0.9	0.5	0.5	0.6
Difficulty Walking	7.7	7.5	7.6	8.8	8.2	9.5	8.4	8.2	8.3
Vertigo	11.5	12.4	11.3	14.5	12.1	14.1	12.4	12.4	12.1
Ambulance Transport	6.9	4.7	6.8	12.4	7.3	12.5	7.3	5.4	7.5
Cancer Screenings	41.0	39.2	40.9	39.3	40.4	35.5	40.6	43.6	40.1
Hospital Admissions	13.2	10.1	13.2	25.5	13.6	23.8	13.4	10.0	14.3

^a^Number of number of distinct drug prescriptions filled in the 14 days prior to antihypertensive initiation

^b^Medication indicated to be associated with fracture risk according to the 2015 Beers Medication Guideline (By the American Geriatrics Society 2015 Beers Criteria Update Expert Panel 2015)

Prevalence of baseline characteristics was identified 12 months prior to initiation of antihypertensive monotherapy

Race was missing for a total of 147 beneficiaries and these were excluded from the SMR weighted analysis

Standardized mortality ratio weighting (SMRW) was used to weight beneficiaries of each drug class to achieve the same baseline covariate distribution as beneficiaries receiving an ACE. Beneficiaries initiating with ACEs were assigned a weight of 1 and all others were assigned a weight that was the ratio of the propensity score to 1 minus the propensity score

*ACE* Angiotensin converting enzyme inhibitors, *ARB* angiotensin receptor blockers, *BB* beta blockers, *CCB* calcium channel blockers, or *THZ* thiazide diuretics

During the first year after initiation of antihypertensive monotherapy, beneficiaries experienced 4430 incident non-vertebral fractures over 115,991 person-years (rate = 382 per 10,000 person-years, 95%CI: 371–393). Fractures most commonly occurred at the hip (79%), foot (17%), radius (15%), and hand (14%). Just over three-quarters of fractures resulted in a single-bone break (77%).

Rates of incident fracture varied according to antihypertensive class and by time since initiation (Table 2, Additional file 1: Figure S2). During the first 14 days, beneficiaries who initiated with THZs (438 per 10,000 person-years, 95%CI: 294–628) and BBs (410 per 10,000 person-years, 95%CI: 314–526) had the highest rate of fractures. Beneficiaries initiating with CCBs had the highest rate of fractures during the 15–365 days after initiation (435 per 10,000 person-years, 95%CI: 404–468), but a low rate in the first 14 days (383 per 10,000 person-years, 95%CI: 258–550). Initiators of ARBs had the lowest rate of fractures during the initial 14 days (333 per 10,000 person-years, 95%CI: 190–546) and during the 15–365 days after initiation (321 per 10,000 person-years, 95%CI: 287–358).

**Table 2:** Rates and hazard ratios of incident fractures within the first year of initiating antihypertensive monotherapy

Drug Class	1–14 days after initiation				15–365 days after initiation			
	# Fractures	P-Yrs	Rate Per 10,000 P-Yrs (95% CI)	SMRW HR (95% CI)	# Fractures	P-Yrs	Rate Per 10,000 P-Yrs (95% CI)	SMRW HR (95% CI)
ACE	54	1539	351 (266, 454)	ref	1271	36,618	347 (328, 367)	ref
ARB	14	420	333 (190, 546)	0.92 (0.49, 1.75)	322	10,032	321 (287, 358)	0.96 (0.84, 1.09)
BB	58	1416	410 (314, 526)	1.00 (0.65, 1.54)	1375	33,449	411 (390, 433)	1.09 (1.00, 1.19)
CCB	27	705	383 (258, 550)	0.82 (0.50, 1.36)	720	16,540	435 (404, 468)	1.11 (1.00, 1.24)
THZ	27	617	438 (294, 628)	1.40 (0.78, 2.52)	562	14,656	384 (353, 416)	1.02 (0.90, 1.15)

*P-Yrs* person-years (calculated by dividing the total number of follow-up days by 365.25)

*SMRW* Standardized mortality ratio weight, calculated adjusting for all baseline covariates

Incident fracture rates and corresponding 95% CIs were defined as the total number of incident fractures by the total P-Yrs at risk. Hazard ratios (*HRs*) and 95% confidence intervals (*CIs*) were calculated using SMR weighted Cox proportional hazard models using a ‘first-treatment-carried-forward’ analysis design. CIs were calculated using robust standard errors to account for the SMRWs

*ACE* Angiotensin converting enzyme inhibitors, *ARB* angiotensin receptor blockers, *BB* beta blockers, *CCB* calcium channel blockers, or *THZ* thiazide diuretics

After controlling for differences in baseline characteristics, beneficiaries who initiated with THZs had the highest rate of fractures in the first 14 days after initiation compared to beneficiaries who initiated with ACEs (SMR-HR: 1.40, 95%CI: 0.78–2.52). After the first 14 days, beneficiaries who initiated with CCBs (SMR-HR: 1.11, 95%CI: 1.00–1.24) and BBs (SMR-HR: 1.09, 95% CI: 1.00–1.19) had slightly higher fractures rates compared to the beneficiaries who initiated with ACEs.

When we stratified results according to fracture location (probable low BMD fractures vs. normal BMD fractures), results were similar for all the antihypertensive classes except THZs (Table 3). During the 1 year following initiation, beneficiaries who initiated with THZs had a lower hazard ratio of probable low BMD fractures (SMR-HR: 0.85, 95%CI: 0.68–1.06), but a slightly higher hazard ratio of normal BMD fractures (SMR-HR: 1.12, 95%CI: 0.98–1.29) compared to beneficiaries who initiated with ACEs.

**Table 3:** Rates of probable low and normal bone mineral density fractures within 1 year of initiating antihypertensive monotherapy

Drug Class	Probable Low Bone Mineral Density Fractures				Normal Bone Mineral Density Fractures			
	# Fractures	P-Yrs	Rate Per 10,000 P-Yrs (95% CI)	SMRW HR (95% CI)	# Fractures	P-Yrs	Rate Per 10,000 P-Yrs (95% CI)	SMRW HR (95% CI)
ACE	424	38,157	111 (101, 122)	ref	901	38,157	236 (221, 252)	ref
ARB	99	10,452	95 (77, 115)	0.93 (0.74, 1.17)	237	10,452	227 (199, 257)	0.97 (0.84, 1.13)
BB	464	34,864	133 (121, 146)	1.08 (0.93, 1.26)	969	34,864	278 (261, 296)	1.09 (0.98, 1.21)
CCB	280	17,245	162 (144, 182)	1.13 (0.95, 1.35)	467	17,245	271 (274, 296)	1.09 (0.96, 1.24)
THZ	171	15,273	112 (96, 130)	0.85 (0.68, 1.06)	418	15,273	274 (248, 301)	1.12 (0.98, 1.29)

*P-Yrs* person-years (calculated by dividing the total number of follow-up days by 365.25)

*SMRW* Standardized mortality ratio weight, calculated adjusting for all baseline covariates

Incident fracture rates and corresponding 95% CIs were defined as the total number of incident fractures by the total P-Yrs at risk. Hazard ratios (*HRs*) and 95% confidence intervals (*CIs*) were calculated using SMR weighted Cox proportional hazard models using a ‘first-treatment-carried-forward’ analysis design. CIs were calculated using robust standard errors to account for the SMRWs”

Probable low bone mineral density fractures included hip and radius fractures. All other non-vertebral fractures were defined as normal bone mineral density fractures

*ACE* Angiotensin converting enzyme inhibitors, *ARB* angiotensin receptor blockers, *BB* beta blockers, *CCB* calcium channel blockers, or *THZ* thiazide diuretics

**Fig. 1:**
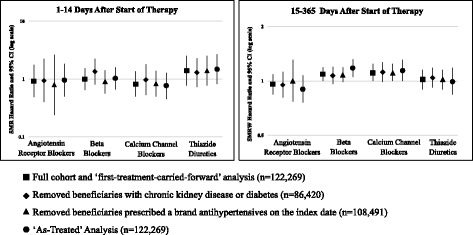
Results of sensitivity analyses comparing the rates of incident fractures according to antihypertensive class. The reference group for the standardized mortality ratio (SMR) weights was beneficiaries who initiated with angiotensin converting enzyme inhibitors

In sensitivity analyses, results were similar when we 1) used an ‘as-treated’ analysis, 2) excluded beneficiaries with a previous diagnosis of chronic kidney disease or diabetes, and 3) excluded beneficiaries who initiated with brand antihypertensive drugs (Fig. 1, Additional file 1: Table S4). When we extended the initial follow-up period to 30 days, beneficiaries who initiated with THZs (SMR-HR: 1.15, 95%CI: 0.75–1.76) and BBs (SMR-HR: 1.36, 95%CI: 1.01–1.83) still had the highest rate of fractures compared to beneficiaries who initiated with other antihypertensive classes (results not shown).

## Discussion

We found incident fracture rates in the year following initiation of antihypertensive therapy differ depending on antihypertensive class, and these patterns were affected by the time since initiation. Medicare beneficiaries who initiated with THZs or BBs had slightly higher fracture rates during the first 2 weeks compared to beneficiaries who initiated with other antihypertensives. However, during the first year beneficiaries who initiated with CCBs had the highest fracture rate. Similar to previous research, we found beneficiaries who initiated with ACEs or ARBs had the lowest rate of fractures (Butt and Harvey 2015; Choi et al. 2015; Wong et al. 2013).

We found older adults initiating with THZs or BBs had the highest fracture rates during the first 2 weeks following initiation. Similar to our results, Berry et al. found that THZ initiators had an increased odds of hip fracture in the first days following initiation compared to periods of no use (Berry et al. 2013). Butt et al. found that the use of any antihypertensive was associated with an increased risk of hip fracture in the first 45 days following initiation, and that this risk was most elevated for older adults initiating with ACEs (Butt et al. 2012). However, Ruths et al. found that only loop diuretics were associated with an initial increased risk of hip fractures during the first 2 weeks of use when comparing the initial fracture risk among antihypertensive class (Ruths et al. 2015). This initial increase in fracture rates may be due to orthostatic hypotension. Among the classes of antihypertensives, THZs are most strongly associated with orthostatic hypotension (Zia et al. 2015). Given that orthostatic hypotension can be asymptomatic (Butt and Harvey 2015; Gupta 2012), results suggest that clinicians and older adults need to be aware of this potential increased risk of fractures when starting antihypertensive therapy, especially when starting therapy with THZs.

Gangavati et al. found that the risk of falls associated with orthostatic hypotension was lower among older adults with controlled hypertension compared to older adults with uncontrolled hypertension (Gangavati et al. 2011). This suggests that increases in fracture rates once hypertension is controlled may be due to other mechanisms besides orthostatic hypotension. One mechanism that may influence the association between antihypertensives and fractures could be antihypertensives interactions with BMD (Berry and Kiel 2014; Ghosh and Majumdar 2014). We found older adults initiating with THZs had a decreased rate of probable low BMD (hip and radius) fractures in the year following initiation. THZs can impact the risk of low BMD fractures by promoting osteoblast activity and reducing calcium urinary excretion (Ghosh and Majumdar 2014; Aung and Htay 2011). A 2011 Cochrane review found that THZs were associated with as much as a 24% reduction in hip fractures when comparing THZ users vs. non-users (Aung and Htay 2011). Results suggest older adults at elevated risk of fractures may potentially benefit from receiving THZs compared to other classes of antihypertensives. However, research featuring clinical BMD measurements is needed to confirm these findings.

Older adults initiating with CCBs had slightly elevated fracture rates compared with ACE initiators. Previous studies have found inconsistent results regarding the association between CCBs and fractures. Ruths et al. found that CCBs were associated with a decreased risk of hip fractures when comparing periods of use and non-use (Ruths et al. 2015). However, this study was limited to hip fractures and results were unadjusted for comorbidities associated with fractures (Ruths et al. 2015). In another study, Choi et al. found that compared to non-users of antihypertensives, adults prescribed CCBs had a slightly elevated rate of non-vertebral fractures (Choi et al. 2015). In our study, beneficiaries who initiated with CCBs were frailer than beneficiaries who initiated with other antihypertensives. Although we included frailty predictors in the propensity scores, we cannot eliminate the possibility that residual confounding remained after adjustment, given that frailty is multi-dimensional and is difficult to capture with claims data alone (Fried et al. 2004).

Despite conducting sensitivity analyses, this study does have limitations. First, results may be subject to residual confounding. We used SMR weights to limit confounding by indication but it is possible that residual confounding remained due to physical activity, visual impairment, baseline BMD, and alcohol use. Some research suggests removing outlier SMR weights to better control for residual confounding. However, we did not exclude outlier SMR weights since excluding these observations resulted in little difference in the results, suggesting that variations in weights were most likely random. Second, fracture effects observed in our study may have been influenced by other factors such as concurrent medication use, other chronic conditions, or previously undiagnosed hypertension. However, results remained the same in sensitivity analyses excluding beneficiaries with diabetes, chronic kidney disease, and those previously taking loop diuretics suggesting that any residual confounding due to side effects from other chronic conditions or medications is minimal. We were unable to distinguish between newly diagnosed hypertension vs. uncontrolled hypertension treated for the first time using claims data. It is possible that beneficiaries were at elevated risk of fracture due to uncontrolled hypertension prior to starting antihypertensive medication. Third, despite controlling for race in propensity scores, it is likely that we were unable to control for all possible race/ethnicity effects due to limitations in the Medicare race/ethnicity data (McBean 2004). Given that the majority of our study population was White, more research is needed featuring more diverse ethnic/racial population to see if the same results would hold true across racial populations. Fourth, our results are limited to the 1 year period following initiation and only included older adults initiating antihypertensives between 2008 and 2011. Antihypertensives impact BMD in as little as 5–8 weeks in animal studies (Birocale et al. 2016; Kang et al. 2013), however less is known about the time it takes for antihypertensives to have clinical BMD effects in humans (Ghosh and Majumdar 2014). One year may not have been long enough to identify all possible BMD affects. Additionally, it is possible that since the time of our study new hypertensive medications have been added to the individual drug classes, however the overall effects of the drug classes would likely remain unchanged. Lastly, our results did not take into account antihypertensive dose. Previous research suggests that the relationship between antihypertensives and fractures is linearly associated with increasing dose (Butt and Harvey 2015; Lipsitz et al. 2015). Results may be underestimated for older adults on higher doses of antihypertensives.

## Conclusions

It is important that researchers and clinicians identify modifiable factors that may reduce the risk of fractures among older adults. We found certain classes of antihypertensive medications may impact the rate of fractures in older adults. Specifically, we found slightly increased fracture rates among older adults initiating with THZs and BBs during the first 2 weeks after initiation. Results suggest older adults taking these medications should be aware of this possible increased risk of fractures, particularly in the first few weeks after starting therapy. Also, we found older adults initiating with ACEs and ARBs had slightly lower fracture rates after initiation. However, given that our results may be due to chance variation in effects, more research is needed to verify if these same results hold true across various racial/ethnic populations and across longer periods of follow-up. Results suggest that when deciding upon antihypertensive therapy, clinicians may want to consider possible fracture risks when choosing between antihypertensive drug classes.

## Additional file

Additional file 1: Table S1. List of antihypertensive drugs included in study. **Table S2.** Definitions of fractures identified in Medicare Claims. **Table S3.** List of chronic conditions and frailty indicators identified using ICD-9 or CPT Codes. **Table S4.** Sensitivity analysis results examining the rates of incident non-vertebral fractures in the first year after initiation among Medicare beneficiaries initiating antihypertensive monotherapy from 2008 to 2011 according to duration of use. **Figure S1.** Eligibility criteria for Medicare beneficiaries initiating antihypertensive monotherapy between 2008 and 2011. **Figure S2.** Weighted Kaplan-Meier curves for incident fractures according duration of use and antihypertensive drug class. (DOCX 134 kb)

## Abbreviations

ACE: Angiotensin-converting enzyme inhibitors
ARB: Angiotensin-receptor blockers
BB: Beta blockers
BMD: Bone mineral density
CCB: Calcium channel blockers
CI: Confidence interval
HR: Hazard ratio
LIS: Low income subsidy
PS: Propensity score
SMR: Standardized mortality ratio
THZ: Thiazide diuretics
