# Supplementary material for: Initiation of antihypertensive monotherapy and incident fractures among Medicare beneficiaries
Source: Inj Epidemiol. 2017 Oct 18;4:27. doi: 10.1186/s40621-017-0125-8 (PMC5645300; doi:10.1186/s40621-017-0125-8)
Supplement: Additional file 1: Table S1. — List of antihypertensive drugs included in study. Table S2. Definitions of fractures identified in Medicare Claims. Table S3. List of chronic conditions and frailty indicators identified using ICD-9 or CPT Codes. Table S4. Sensitivity analysis results examining the rates of incident non-vertebral fractures in the first year after initiation among Medicare beneficiaries initiating antihypertensive monotherapy from 2008 to 2011 according to duration of use. Figure S1. Eligibility criteria for Medicare beneficiaries initiating antihypertensive monotherapy between 2008 and 2011. Figure S2. Weighted Kaplan-Meier curves for incident fractures according duration of use and antihypertensive drug class. (DOCX 134 kb) [file 40621_2017_125_MOESM1_ESM.docx]

Additional file 1

| Table S1: List of antihypertensive drugs included in study | |
| --- | --- |
| **Drug Name** | **Generic Drug Names** |
| *ARBs* | Azilsartan, Candesartan, Eprosartan, Fimasartan, Irbesartan, Losartan, Olmesartan, Tasosartan, Telmisartan, Valsartan |
|  |  |
| *ACEs* | Benazepril, Captopril, Cilazapril, Delapril, Enalapril, Fosinopril, Imidapril, Lisinopril, Moexipril, Perindopril, Quinapril, Ramipril, Spirapril, Temocapril, Trandolapril, Zofenopril |
|  |  |
| *CCBs* | Amlodipine, Barnidipine, Benidipine, Bepridil, Clinidipine, Clevidipine, Diltiazem, Felodipine, Fendiline, Gallopamil, Isradipine, Lacidipine, Lercanidipine, Lidoflazine, Manidipine, Nicardipine, Nifedipine, Nilvadipine, Nimodipine, Nisoldipine, Nitrendipine, Perhexiline, Verapamil |
|  |  |
| *BBs* | Acebutolol, Alprenolol, Atenolol, Betaxolol, Bevantolol, Bisoprolol, Bopindolol, Bupranolol, Carteolol, Carvedilol, Celiprolol, Cloranolol, Epanolol, Esmolol, Labetalol, Mepindolol, Metipranolol, Metoprolol, Nadolol, Nebivolol, Netoprolol, Oxprenolol, Penbutolol, Pindolol, Practolol, Propranolol, Sotalol, Talinolol, Tertatolol, Timolol |
| *THZs* | Bendroflumethiazide, Chlorothiazide, Cyclopenthiazide, Cyclothiazide, Hydrochlorothiazide, Hydroflumethiazide, Mebutizide, Methyclothiazide, Polythiazide, Thiazide, Trichlormethiazide |
| Drugs were identified by generic drug name and National Drug Codes (NDC) in Medicare Part D data.  Angiotensin converting enzyme inhibitors (ACE), angiotensin receptor blockers (ARB), beta blockers (BB), calcium channel blockers (CCB), or thiazide diuretics (THZ). | |

| Table S2: Definitions of fractures identified in Medicare Claims | | | |
| --- | --- | --- | --- |
| **Fracture Type** | **ICD-9 Diagnosis Codes** | **ICD-9 Procedure Codes** | **Current Procedure Terminology (CPT) Codes** |
| Hip | 820.x, | 7855, 7905, 7915, 7925, 7935, 7965, 8161, 8162 | 27125-27127, 27230, 27232, 27234-27236, 27238, 27240, 27242, 27244, 27246, 27248, 27130, 27131, 29010, 29015, 29020, 29025, 29035, 29040, 29044, 29046, 29035, 29325, 29345, 29355, 29358, 29365, 29505, 29520, 29799, 73500, 73510, 73520, 73530, 73550 |
| Radius | 813.x, | 7853, 7902, 7912, 7922, 7932, 7962 | 24620, 24625, 24635, 24650, 24655, 24660, 24665, 24666, 24670, 24675, 24680, 24685, 25500, 25505, 25510, 25515, 25530, 25535, 25540, 25545, 25560, 25565, 25570, 25575, 25600, 25605, 25610. 25611, 25615, 25620, 25650, 24580-24581, 24583, 24585-24588, 29065, 29075, 29085, 29105, 29125, 29126, 29799, 73070, 73080, 73090, 73100, 73110 |
| Humerus | 812.x, | 7852, 7901, 7911, 7921, 7931, 7961 | 23600, 23605, 23610, 23615, 23620, 23625, 23630, 23665, 23670, 23675, 23680, 24500, 24505, 24506, 24510, 24515, 24530, 24531, 24535, 24536, 24538, 24540, 24542, 24545, 24560, 24565, 24570, 24575-24581, 24583, 24585-24588, 29035, 29040, 29044, 29046, 29065, 29105, 29799, 73020, 73030, 73050, 73060, 73070, 73080 |
| Rib | 8070, 8071, 8072, 8073, 8074 |  | 21800, 21805, 21810, 21820, 21825, 29010, 29015, 29020, 29025, 29035, 29040, 29044, 29046, 29200, 71100, 71101, 71110, 71111, 71120, 71130 |
| Pelvis | 8056, 8057, 8066, 8067, 808.x |  | 27190-27192, 27200, 27202, 27210-27212, 27214, 27220, 27222, 27224, 27225, 27120, 27122, 27130, 27131-27132, 72010, 72020, 72100, 72110, 72114, 72120, 72170, 72190, 72200, 72202, 72220, 73500, 73510, 73520, 73530 |
| Ankle | 824.x |  | 27760, 27762, 27764, 27766, 27786, 27788, 27790, 27792, 27808, 27810, 27812, 27814, 27816, 27818, 27820, 27822, 27823, 29010, 29015, 29020, 29025, 29345, 29355, 29358, 29365, 29405, 29425, 29505, 29515, 29540, 29799, 70330, 73590, 73600, 73620, 73630 |
| Femoral Shaft | 821.x |  | 27500, 27502, 27504, 27506, 27508, 27510, 27512, 27514, 29010, 29015, 29020, 29025, 29035, 29040, 29044, 29046, 29305, 29325, 29345, 29355, 29348, 29365, 29505, 29520, 29799, 73500, 73510, 73520, 73530, 73550 |
| Hand | 814.x-817.x, | 7854, 7903, 7904, 7913, 7914, 7923, 7924, 7933, 7934, 7963, 7964 | 25622, 25624, 25626, 25628, 25630, 25635, 25640, 25645, 25680, 25685, 26600, 26605, 26607, 26610, 26615, 26645, 26650, 26655, 26660, 26665, 26720, 26725, 26727, 26730, 26735, 26740, 26742, 26743, 26744, 26746, 26750, 26755-26756, 26760, 26765, 25600, 25605, 25610, 25611, 25615, 25620, 25650, 29035, 29075, 29085, 29105, 29125, 29126, 29130, 29131, 29799, 73100, 73110, 73120, 73130, 73140 |
| Tibia | 823.x, | 7857, 7906, 7916, 7926, 7936, 7966 | 27530, 27532, 27534, 27536-27538, 27540, 27750, 27752, 27754, 27756, 27758, 27780, 27781-27782, 27784, 27800, 27802, 27804, 27806, 29010, 29015, 29020, 29025, 29345, 29355, 29358, 29365, 29405, 29425, 29505, 29515, 29799, 73560, 73562, 73564, 73590, 73600, 73610 |
| Skull/Face | 800-804.x | 767 | 21300, 21310, 21315, 21320, 21325, 21330, 21335, 21334-21340, 21345-21347, 21350, 21355, 21360, 21365, 21380, 21385-21387, 21390, 21395-21400, 21401, 21406, 21407, 21420-21422, 21431, 21432-21433, 21435, 21440, 21445, 21450, 21451-21455, 21461, 21462, 21465, 24170, 21495, 70230, 70231, 70250, 70260, |
| Foot | 825.x-826.x, | 7858, 7907, 7908, 7917, 7918, 7927, 7928, 7938, 7967, 7968 | 28400, 28405-28406, 28410, 28415, 28420, 28430, 28415, 28450, 28430, 28435, 28436, 28440, 28445, 28450, 28470, 28475-28476, 28480, 28485, 28490, 28495-28496, 28500, 28505, 28510, 28515, 28520, 28525, 29405, 29425, 29505, 29515, 29550, 29580, 73600, 73620, 73630, 73650, 73660 |
| Clavicle | 810.x-811.x |  | 23500, 23505, 23510, 23515, 23570, 23575, 23585, 29010, 29015, 29020, 29025, 29035, 29040, 29044, 29046, 29049, 29055, 29058, 29065, 29105, 29240, 73000, 73010, 73020, 73030, 73050, 73060 |
| Patella | 822.x | 7856 | 27520, 27522, 27534, 29010, 29015, 29020, 29025, 29035, 29040, 29044, 29046, 29345, 29355, 29358, 29365, 29355, 29358, 29365, 29435, 29505, 29530, 73550, 73560, 73562, 73564, 73590 |
| Fractures were identified using validated diagnosis codes and procedure codes found in Medicare Parts A and B (Ray, Griffin, Fought, & Adams, 1992). Fractures included in the study had an incident diagnosis code with a corresponding procedure code within 7 days of the diagnosis. | | | |

| Table S3: List of chronic conditions and frailty indicators identified using ICD-9 or CPT Codes | |
| --- | --- |
| **Covariate** | **Code (includes ICD-9 and CPT Codes)** |
| Ambulance Transfer | A0426, A0427, A0428, A0429, A0999 |
| Alzheimer’s Disease | 3310 |
| Arrhythmia | 4270, 4271, 4272, 42731, 42732, 42741, 42742, 4275, 42760, 42761, 42769, 42781, 42789, 4279 |
| Cancer Screen | V760, V761, V7610, V7611, V7612, V7619, V762, V763, V7641, V7642, V7643, V7644, V7645, V7646, V7647, V7649, V7650, V7651, V7652, V768, V7681, V7689, V769 |
| Chronic Kidney Disease | 2504, 25040, 25041, 25042, 25043, 27410, 403, 4039, 40390, 404, 4040, 40400, 40401, 40402, 40403, 4041, 40410-40413, 4049, 40490, 40491, 40492, 40493, 4401, 4421, 5724, 580, 5800, 5804, 5808, 58081, 58089, 5809, 581, 5810-5813, 5818, 58181, 58189, 5819, 582, 5820, 5821, 5822, 5824, 5828, 58281, 58289, 5829, 583, 5830, 5831, 5832, 5834, 5836, 5837, 5838, 58381, 58389, 5839, 584, 5845-5849, 585, 5851-5856, 586, 587, 5930- 5937, 59370-59373, 5938, 59381, 59382, 59389, 5939, 753, 7530 ,7533, 7912, 7913, 86600, 86601, 8661, 86610-86613 |
| Dementia | 290, 2900, 29010, 29011, 29012, 29013, 29020, 29021, 2903, 2904, 29041, 29042, 29043, 2908, 2909, 294, 2940, 2941, 29410, 29411, 29420, 29421, 2948, 2949 |
| Difficulty Walking | 7197, 71970, 71975, 71976, 71977, 71978, 71979, 7812, 7813 |
| Diabetes | 2500, 25000, 25002, 25010, 25012, 2502, 25020, 25022, 2503, 25030, 25032, 2504, 25040, 25042, 2505, 25050, 25052, 2506, 25060, 25062, 2507, 25070, 25072, 2508, 25080, 2509, 25090, 25092 |
| Dyslipidemia | 272, 2720, 2721, 2722, 2723, 2724, 2725, 2726, 2727, 2728, 2729 |
| Hospital Bed Use | E0250, E0251, E0255, E0256, E0260, E0261, E0265, E0266, E0270, E0290, E0291, E0292, E0293, E0294, E0295, E0296, E0297, E0301, E0302, E0303, E0304, E0316 |
| Home Oxygen Use | E0431, E0433, E0434, E0435, E0439, E0441, E0442, E0443, E1390, E1391, E1392 |
| Hypertension | 4010, 4011, 4019 |
| Myocardial Infarction | 410, 4100 ,41001, 4101, 41011, 4102, 41021, 4103, 41031, 4104, 41041, 4105, 41051, 4106, 41061, 4107, 41071, 4108, 41081, 4109, 41091 |
| Obesity | 27800, 27801, 27803 |
| Orthostatic Hypotension | 4580 |
| Osteoarthritis | 71500, 71504, 71509, 71510-71518, 71520-71528, 71530-71538, 71580, 71589 |
| Osteoporosis | 73300, 73301, 73302, 73303, 73309 |
| Parkinson’s Disease | 332, 3320, 3321 |
| Rheumatoid Arthritis | 7140, 7142, 71430, 71431, 71432 |
| Stroke | 430, 431, 432, 4320, 4321, 4329, 4330, 43300, 43301, 4331, 43310, 43311, 4332, 43320, 43321, 4333, 43330, 43331, 4338, 43380, 43381, 4339, 43390, 43391, 4340, 43400, 43401, 4341, 43410, 43411, 4349, 43490, 43491, 435, 4350, 4351, 4352, 4353, 4358, 4359, 436, 4370-4379, 438, 4380, 43810, 43811-43814, 43819, 43820, 43821, 43822, 43830, 43831, 43832, 43840, 43841, 43842, 43850, 43851, 43852, 43853, 4386, 4387, 43881-43885, 43889, 4389 |
| Syncope | 7802 |
| Urinary Incontinence | 7883, 78830, 78831, 78832, 78833, 78834, 78835, 78836, 78837, 78838, 78839 |
| Walker or Wheelchair Use | E0130, E0135, E0140, E0141, E0143, E0144, E0147, E0148. E0149, E0154, E0155, E0156, E0157, E0158, E1050, E1060, E1070, E1083, E1084, E1085, E1086, E1087, E1088, E1089, E1090, E1091, E1092, E1093, E1100, E1140, E1150, E1160, E1161, E1170, K0001, K0002, K0003, K0004, K0005, K0006, K0007, K0008, K0009 |
|  |  |
|  |  |
|  |  |
| Vertigo | 3860, 38600, 38601, 38602, 38603, 38604, 3861, 38610, 38611, 38612, 38619, 3862, 3863, 38630, 38631, 38632, 38633, 38634, 38635, 3864, 38640, 38641, 38642, 38643, 38648, 3865, 38650, 38651, 38652, 38653, 38654, 38655, 38656, 38658, 3868, 3869, 43885, 7804 |
| Covariates were identified using Medicare Parts A and B data using International Classification of Diseases, Ninth Revision (ICD-9) and Current Procedural Terminology Codes (CPT). | |

| Table S4: Sensitivity analysis results examining the rates of incident non-vertebral fractures in the first year after initiation among Medicare beneficiaries initiating antihypertensive monotherapy from 2008-2011 according to duration of use | | | | | | | | |
| --- | --- | --- | --- | --- | --- | --- | --- | --- |
|  | **1-14 Days After Initiation** | | | | **15-365 Days After Initiation** | | | |
| Drug Class | # Fractures | P-Yrs | Rate Per 10,000  P-Yrs (95% CI) | SMRW HR (95% CI) | # Fractures | P-Yrs | Rate per 10,000  P-Yrs (95% CI) | SMRW HR (95% CI) |
| *Results when using an ‘As-Treated’ analysis (n=122,629)* | | | | | | | | |
| ACE | 52 | 1,502 | 346 (261-451) | ref | 735 | 21,726 | 338 (315-363) | ref |
| ARB | 14 | 410 | 342 (194-559) | 0.96 (0.50, 1.82) | 177 | 5,973 | 296 (255-343) | 0.90 (0.76, 1.07) |
| BB | 55 | 1,373 | 401 (305-518) | 1.03 (0.67, 1.60) | 842 | 19,802 | 425 (397-455) | 1.18 (1.05, 1.32) |
| CCB | 24 | 679 | 354 (232-518) | 0.77 (0.45, 1.29) | 432 | 9,534 | 453 (412-497) | 1.14 (1.00, 1.31) |
| THZ | 27 | 595 | 454 (305-651) | 1.48 (0.82, 2.67) | 277 | 7,932 | 349 (310-392) | 0.99 (0.84, 1.18) |
| *Results when excluding beneficiaries with chronic kidney disease or diabetes (n=86,420)* | | | | | | | | |
| ACE | 32 | 979 | 327 (227-456) | ref | 774 | 23,373 | 331 (308-355) | ref |
| ARB | <11 | NR | 300 (139-569) | 0.94 (0.40, 2.22) | 196 | 6,398 | 306 (266-352) | 0.95 (0.81, 1.12) |
| BB | 45 | 1,045 | 431 (318-571) | 1.35 (0.81, 2.26) | 994 | 24,799 | 401 (377-426) | 1.07 (0.96, 1.20) |
| CCB | 19 | 513 | 370 (230-568) | 0.97 (0.53, 1.81) | 517 | 12,116 | 427 (391-465) | 1.12 (0.99, 1.27) |
| THZ | 22 | 505 | 436 (280-649) | 1.30 (0.74, 2.30) | 456 | 12,031 | 379 (345-415) | 1.04 (0.92, 1.18) |
| *Results when excluding beneficiaries who initiated therapy with brand antihypertensive medications (n=108,491)* | | | | | | | | |
| ACE | 54 | 1,529 | 353 (268-457) | ref | 1,263 | 36,373 | 347 (329-367) | ref |
| ARB | <11 | NR | 370 (94-1,008) | 0.80 (0.24, 2.62) | 66 | 1,942 | 340 (265-430) | 1.00 (0.77, 1.31) |
| BB | 51 | 1,294 | 394 (297-514) | 0.90 (0.57, 1.41) | 1,257 | 30,554 | 411 (389-435) | 1.08 (0.99, 1.19) |
| CCB | 25 | 637 | 393 (260-571) | 0.83 (0.50, 1.39) | 655 | 14,954 | 438 (405-473) | 1.11 (1.00, 1.24) |
| THZ | 27 | 614 | 440 (296-631) | 1.40 (0.78, 2.52) | 559 | 14,583 | 383 (353-416) | 1.02 (0.90, 1.15) |
| *Results excluding beneficiaries who had a loop diuretic filled during baseline (n=115,297)* | | | | | | | | |
| ACE | 50 | 1,451 | 345 (259, 451) | ref | 1,153 | 34,561 | 334 (315, 353) | ref |
| ARB | 12 | 395 | 304 (165, 517) | 0.83 (0.41, 1.67) | 305 | 9,454 | 323 (288, 361) | 1.00 (0.88, 1.15) |
| BB | 54 | 1,317 | 410 (311, 531) | 1.06 (0.68, 1.64) | 1,235 | 31,182 | 396 (374, 419) | 1.08 (0.98, 1.18) |
| CCB | 21 | 660 | 318 (202, 478) | 0.76 (0.44, 1.31) | 668 | 15,525 | 430 (399, 464) | 1.14 (1.02, 1.27) |
| THZ | 25 | 594 | 421 (278, 612) | 1.32 (0.71, 2.44) | 528 | 14,135 | 374 (343, 406) | 1.01 (0.89, 1.14) |
| P-Yrs= person-years of follow (calculated by dividing the total number of follow-up days by 365.25)  SMRW= Standardized mortality ratio weight  Hazard ratios (HR’s) and 95% confidence intervals (CIs) are adjusted for SMRWs that were calculated using all the baseline covariates.  NR= non-reportable  Angiotensin converting enzyme inhibitors (ACE), angiotensin receptor blockers (ARB), beta blockers (BB), calcium channel blockers (CCB), or thiazide diuretics (THZ) | | | | | | | | |


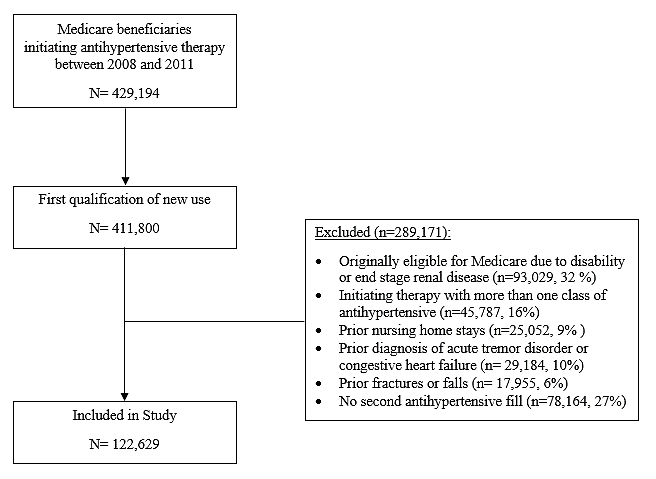


Figure S1: Eligibility criteria for Medicare beneficiaries initiating antihypertensive monotherapy between 2008-2011.


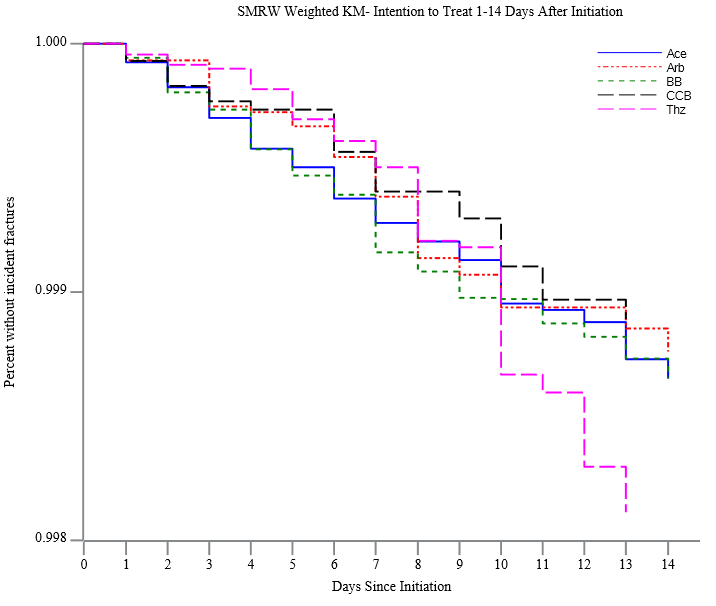


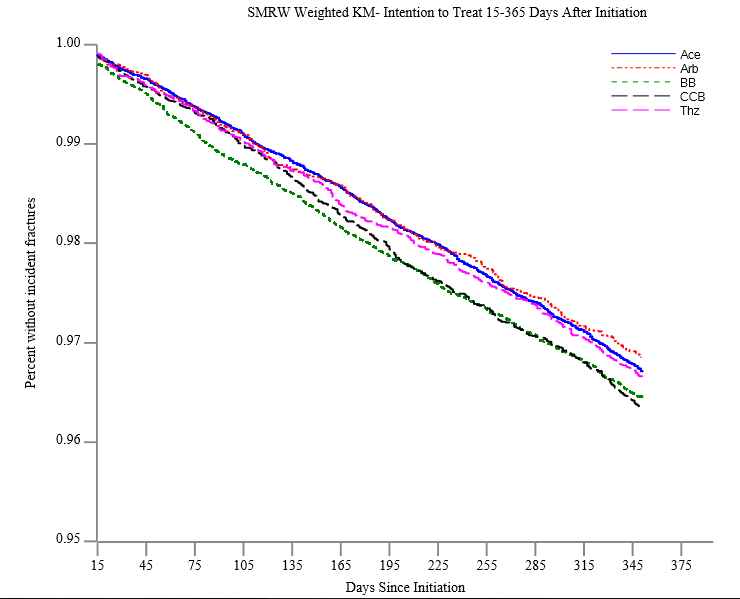


Figure S2: Weighted Kaplan-Meier curves for incident fractures according duration of use and antihypertensive drug class.

Legend: Top figure =1-14 days after initiation. Bottom figure =15-365 days after initiation. Angiotensin converting enzyme inhibitors (ACE), angiotensin receptor blockers (ARB), beta blockers (BB), calcium channel blockers (CCB), or thiazide diuretics (THZ).
